# Assay validity of point-of-care platelet function tests in thrombocytopenic blood samples

**DOI:** 10.11613/BM.2022.020713

**Published:** 2022-06-15

**Authors:** Conrad Lacom, Alexander Tolios, Markus W. Löffler, Beate Eichelberger, Peter Quehenberger, Eva Schaden, Marion Wiegele

**Affiliations:** 1 Department of Anaesthesia, Critical Care and Pain Medicine, Division of General Anaesthesia and Intensive Care Medicine, Medical University of Vienna, Vienna, Austria; 2 Department of Blood Group Serology and Transfusion Medicine, Medical University of Vienna, Vienna, Austria; 3 Center for Physiology and Pharmacology, Institute of Vascular Biology and Thrombosis Research, Medical University of Vienna, Vienna, Austria; 4 Center for Medical Statistics, Informatics, and Intelligent Systems, Institute for Artificial Intelligence and Decision Support, Medical University of Vienna, Vienna, Austria; 5 Department of Immunology, Interfaculty Institute for Cell Biology, University of Tübingen, Tübingen, Germany; 6 Department of General, Visceral and Transplant Surgery, University Hospital Tübingen, Tübingen, Germany; 7 Department of Clinical Pharmacology, University Hospital Tübingen, Tübingen, Germany; 8 Cluster of Excellence iFIT (EXC 2180) “Image-Guided and Functionally Instructed Tumor Therapies”, University of Tübingen, Tübingen, Germany; 9 Department of Laboratory Medicine, Medical University of Vienna, Vienna, Austria

**Keywords:** flow cytometry, platelet function, point-of-care tests, thrombocytopenia

## Abstract

**Introduction:**

Point-of-care (POC) platelet function tests are faster and easier to perform than in-depth assessment by flow cytometry. At low platelet counts, however, POC tests are prone to assess platelet function incorrectly. Lower limits of platelet count required to obtain valid test results were defined and a testing method to facilitate comparability between different tests was established.

**Materials and methods:**

We assessed platelet function in whole blood samples of healthy volunteers at decreasing platelet counts (> 100, 80-100, 50-80, 30-50 and < 30 x10^9^/L) using two POC tests: impedance aggregometry and *in-vitro* bleeding time. Flow cytometry served as the gold standard. The number of platelets needed to reach 50% of the maximum function (ED_50_) and the lower reference limit (ED_ref_) were calculated to define limits of test validity.

**Results:**

The minimal platelet count required for reliable test results was 100 x10^9^/L for impedance aggregometry and *in-vitro* bleeding time but only 30 x10^9^/L for flow cytometry. Comparison of ED_50_ and ED_ref_ showed significantly lower values for flow cytometry than either POC test (P value < 0.05) but no difference between POC tests nor between the used platelet agonists within a test method.

**Conclusion:**

Calculating the ED_50_ and ED_ref_ provides an effective way to compare values from different platelet function assays. Flow cytometry enables correct platelet function testing as long as platelet count is > 30 x10^9^/L whereas impedance aggregometry and *in-vitro* bleeding time are inconsistent unless platelet count is > 100 x10^9^/L.

## Introduction

Platelet function can be rapidly assessed by various point-of-care (POC) tests, such as impedance aggregometry (IA) and *in-vitro* bleeding time (IVBT). Their short turnaround times enable prompt clinical decisions and goal-directed therapy in resuscitation of trauma patients, haemostatic management in the critically ill or in the perioperative and interventional setting. However, normal platelet count is recommended for test performance (*1*-*12*). This often precludes the use of POC tests in the critically ill patient, who may have a low platelet count due to inflammatory processes (sepsis), chemotherapy, extracorporeal therapies, liver failure or platelet consumption following active bleeding or thromboembolic events. If the remaining platelets’ function is preserved, this may lead to inappropriate platelet transfusions or withholding of anticoagulation (*13*-*15*). In thrombocytopenic patients, flow cytometry (FC) is considered to be the gold standard for determination of platelet function but its low availability, complex test performance and long turnaround times limit the clinical utility in rapid decision making (*16*, *17*).

Solid evidence concerning the minimum platelet count required for POC tests on platelet function is scarce and studies either did not include IVBT, differentiated poorly within the thrombocytopenic range or were limited by the methodology used for the blood dilution process (*3*, *9*, *11*). To the best of our knowledge, an intra-individual comparison of samples with reduced platelet counts including IA, IVBT and FC has not yet been performed. The primary goal of this study was therefore to define a minimum platelet count above which valid information on platelet function can be derived from respective tests. Furthermore, we aimed to establish a new model for comparison of different test methods.

## Materials and methods

### Subjects

For this *in-vitro* study, ten healthy volunteers (> 18 years) were recruited among platelet donors scheduled for platelet donation at the Department of Blood Group Serology and Transfusion Medicine at the Medical University of Vienna between November 2018 and July 2019. Prior to inclusion, the medical and bleeding history was assessed according to the Austrian Association of Anaesthetists’ standardized pre-anaesthetic questionnaire to rule out hereditary coagulation disorders and intake of drugs or phytopharmaceuticals known to influence haemostasis (*18*). We did not enrol pregnant women.

Informed consent was obtained from all individuals included in this study. Research complied with all relevant national regulations, and institutional policies and is in accordance with the tenets of the Helsinki Declaration (as revised in 2013), and has been approved by the authors’ Institutional Review Board (Ethikkommission Medizinische Universität Wien) (No. 1468/2017).

### Methods

#### Demographic data and baseline measurements

Recorded demographic data included sex and age. Baseline laboratory assessments on undiluted blood samples comprised haematocrit and platelet count as well as the platelet function tests IA, IVBT and FC.

#### Blood sampling and processing

Prior to the scheduled platelet donation, whole blood (36 mL) was drawn from a peripheral vein into six 3 mL trisodium citrate tubes (3.2%, 9:1 v/v) for IVBT and FC and three 6 mL lithium heparin tubes for IA (both tubes Vacuette Greiner, Kremsmünster, Austria). Blood collections took place between 7:45 and 8:15 a.m. in the non-fasted state and in an alternating order between the two different types of tubes.

After performing an automated complete blood count (DxH 500 haematology analyser, Beckman Coulter, Inc., Brea, USA), blood samples of each individual participant were diluted to yield five different platelet counts (> 100 (*i.e.,* undiluted sample), 80–100, 50–80, 30–50 and < 30 x10^9^/L) using the method described by Bercovitz *et al.* with the following modifications: fresh whole blood was aliquoted and one part was centrifuged for 10 minutes at 300xg to separate the corpuscular part of the red blood cell concentrate (RBCC) from the platelet rich plasma (PRP) and buffy coat (Rotixa 500 RS, Hettic GmbH& Co. KG, Tuttlingen, Germany) (*19*). In a subsequent step the PRP was centrifuged for 10 minutes at 3800xg to obtain platelet poor plasma (PPP) (Heraeu Fresco 17, Thermo Fisher Scientific Inc., Schwerte, Germany). To minimize the number of residual platelets in the RBCC, it was washed using sodium chloride 0.9% (Rotina 380, Hettich GmbH& Co. KG, Tuttlingen, Germany). All centrifugation steps were performed at room temperature.

The final platelet poor whole blood samples (PPWB) were obtained by mixing the obtained RBCC and PPP in the appropriate ratios. Thus, the initial haematocrit and concentration of plasma proteins of whole blood were restored, while platelets had been depleted. It was essential to maintain the initial whole blood’s haematocrit since it is known to affect POC test results (*20*, *21*).

**Figure 1:**
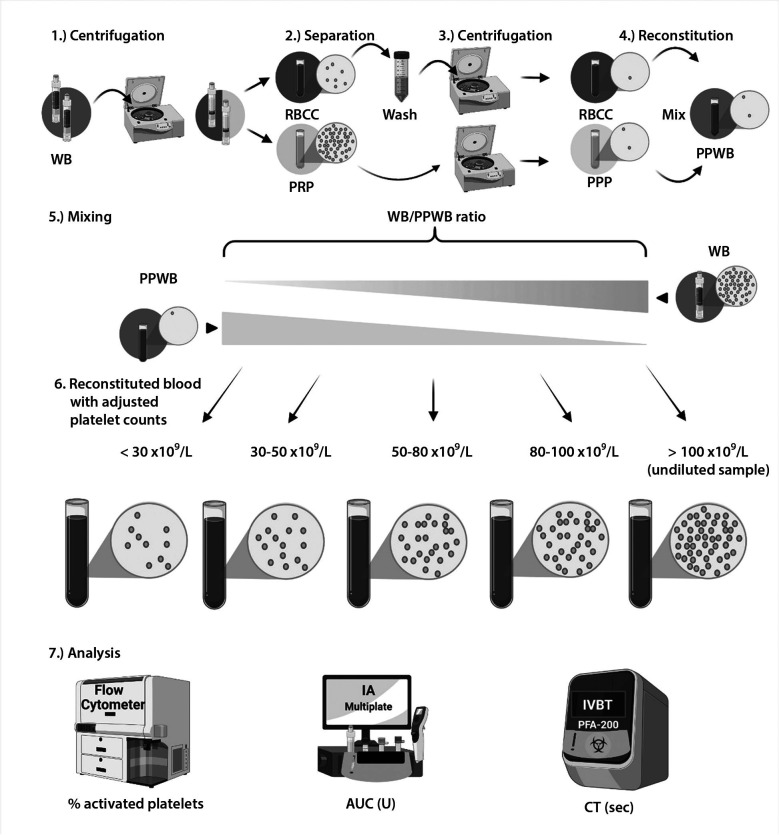
Whole blood dilution was performed in two steps: first by mixing PPP with the RBCC, and followed by a second mixing step with an aliquot of the original sample. To generate the PPWB, the ratio of RBCC to PPP was approximately „1 to (X - 1)", whereas X is the ratio "haematocrit (RBCC) / haematocrit (WB)“, assuming a haematocrit (PPP) of 0. To generate the final samples, the platelet count was approximated by using "Y mL" of WB and "Y x (Z - 1) mL" of PPWB, whereas Y is "the amount of blood required to run all platelet function tests / Z" and Z being defined as the ratio "platelet count (WB) / desired platelet count (final sample). PPP – platelet poor plasma. RBCC – red blood cell concentrate. PPWB – platelet poor whole blood. WB – whole blood.

In a next step, aliquots of the original whole blood with unchanged platelet count (WB) were mixed with the PPWB to obtain the samples with different platelet counts used for analysis. Platelet counts and haematocrit were determined at certain steps along the dilution process to provide quality control (PPP, RBCC, PPWB) as well as for all final samples (DxH 500 haematology analyser, Beckman Coulter, Inc., Brea, USA). Details of blood sample processing can be found in Figure 1.

#### Platelet function testing

The following platelet function tests were run on all five samples (> 100 (*i.e.,* undiluted sample), 80–100, 50–80, 30–50, and < 30 x10^9^/L) of each participant within a maximal time delay of four hours from venepuncture.

#### Impedance aggregometry (IA)

Impedance aggregometry (also referred to as multiple electrode aggregometry) was performed on a multiplate analyser (Roche Diagnostics GmbH, Mannheim, Germany) as described by Calatzis and colleagues (*22*). Platelet aggregation leads to an increase in impedance between two electrodes immersed in a mixture of whole blood and saline. Platelet function is described by an area under the curve (AUC) expressed in Units (U), one Unit corresponding to a defined impedance change *per* minute. 32 µM of thrombin receptor activating peptide (TRAP) (reference ranges for normal platelet function: 92-151 U) (Bachem Holding AG, Bubendorf, Switzerland) and 6.4 µM of adenosine diphosphate (ADP) (reference ranges: 55-117 U) (Roche Diagnostics GmbH, Mannheim, Germany) were used as agonists.

#### In-vitro bleeding time (IVBT)

We assessed IVBT *via* INNOVANCE PFA-200 (Siemens Healthcare Diagnostics Products GmbH, Marburg, Germany). Commercially available, prefabricated cartridges containing collagen/epinephrine (Col/Epi) or collagen/ADP (Col/ADP) as agonists were used. A shorter “closure-time” (CT, given in seconds) referred to increased platelet function with reference ranges of 82-150 sec for the Col/Epi test and 62-100 sec for the Col/ADP test application, according to the manufacturer’s specification. Closure time measurement stopped at a maximum of 300 sec.

#### Flow cytometry (FC)

For FC, citrated whole blood was diluted with phosphate buffered saline (1:10) and incubated with an APC-labeled glycoprotein Ib alpha antibody (anti-CD42b, BD Pharmingen San Jose, USA) as a platelet surface marker (*23*). Platelets were stimulated using either TRAP-6 (14,25 µM) or ADP (1 µM) and stained with a PE-labelled P-selectin antibody (anti-CD62P, Immunotech SAS, Beckman Coulter Inc., Marseille, France) as a platelet activation marker. Established clinical reference values were used as cut-offs (> 63% activated platelets for TRAP and > 42% for ADP) (*23*). Measurements were performed using a BD FACSCanto II (BD Biosciences, San Jose, USA), recording at least 10,000 events *per* sample. Flow cytometry is recommended for platelet function testing in case platelet count is < 100 x10^9^/L and served as the standard method for comparison in this study (*24*, *25*).

### Data processing

To establish comparability between the output of different methods, absolute values of test results were transformed into relative values: from all analysed samples, the overall highest and lowest values measured in both FC and IA were defined to represent 1 or 0, respectively. For IVBT, those values were inverted since high values represent a reduced platelet function. All other values were linearly transformed to represent numbers between 0 and 1.

After data transformation, a sigmoidal fitting function was calculated for each serial dilution (*per* participant and test) as provided by the R-package *drc,* which is a computational library for the analysis of dose-response curves and versatile model fitting and after-fitting functions (*26*). This was used to develop a new model for inter-assay comparison by calculating the platelet count at which each platelet function test reaches a value of 0.5 (representing a measured platelet function half way between the lowest and highest measurement of each participant, termed ED_50_ in accordance with pharmacological studies where this refers to the median effective dose) or a value corresponding to the upper (in case of IVBT) or lower (in case of FC or IA) threshold, considered as not yet pathological in a clinical context (termed the lower reference limit (ED_ref_)). The ED_ref_ is therefore the platelet count, below which the test method gives a result outside of the reference range despite a normal platelet function in the undiluted whole blood sample. Converting thresholds of normal platelet function into relative values (on a scale 0-1, as defined above) gave the following results:

Impedance aggregometry: 0.69 for the TRAP assay and 0.69 for the ADP assay at absolute threshold values 92U and 55U, respectively

*In-vitro* bleeding time: 0.71 for the Col/Epi assay and 0.9 for the Col/ADP assay at absolute threshold values 150 sec and 100 sec, respectively.

Flow cytometry: 0.62 for the TRAP assay and 0.48 for the ADP assay at absolute threshold values 63% and 42%, respectively.

#### Statistical analysis

Statistical analysis was performed with the free and open source software GNU R version 3.5.3 together with additional, optional package libraries as described in the appropriate sections (*26*). Age was presented as median, minimum, and maximum values, platelet count and haematocrit as median values and interquartile range. Median effective dose and ED_ref_ were described using median values. Group comparison was performed using the Wilcoxon-rank-sum-test for unadjusted groups and the chi-square or Fisher’s exact test for comparing categorical variables. Dunn’s Multiple Comparison Test was used as a *post hoc* non-parametric test for group comparisons (*27*). To account for the number of multiple comparisons performed, the Bonferroni-Holm correction was applied accordingly. All P values are results of two-sided tests, and P values < 0.05 were considered statistically significant.

## Results

### Study population

Seven male and three female volunteers with a median age of 31 years (28-55 years) were included in the study. One male individual showing abnormal intrinsic platelet function in baseline measurements despite a negative bleeding history was excluded from further analyses.

### Automated platelet count

Initial median platelet count and haematocrit were 183 (66) x10^9^/L and 0.37 (0.05) L/L, respectively.

Platelet counts were achieved as defined in the methods section while maintaining a haematocrit within a median deviation of ± 2.5% from initial values. Median residual platelet count in PPWB was 16.5 (8.5) x10^9^/L.

### Platelet function tests

#### Impedance aggregometry

**Figure 2:**
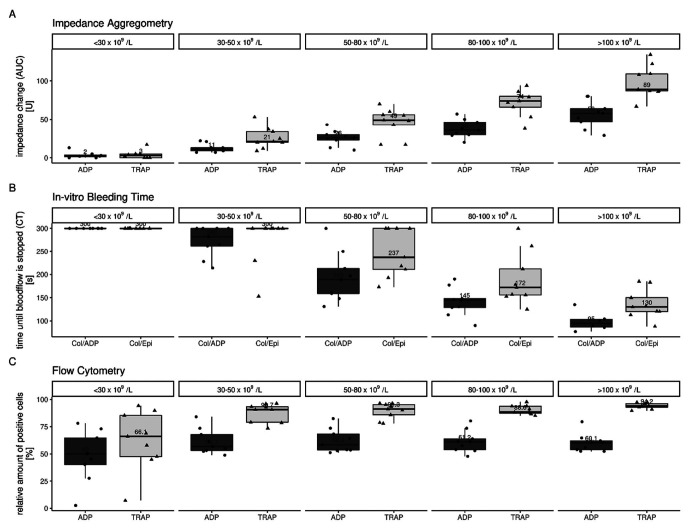
Impact of platelet count on platelet function assessed by a) IA, b) IVBT, and c) FC. The vertical axis depicts the raw platelet function measurements: a) AUC in IA, b) CT in IVBT, and c) percentage of activated platelets in FC. The horizontal axis shows decreasing platelet counts in x10^9^/L from right to left (top of each graph) and the agonist used (bottom of each graph). IA – impedance aggregometry. IVBT – *in-vitro* bleeding time. FC – flow cytometry. AUC – area under the curve. CT – closure time. ADP – adenosine diphosphate. TRAP – thrombin receptor- activating peptide. Col/ADP – collagen/ adenosine diphosphate. Col/Epi – collagen/ epinephrine.

**Figure 3:**
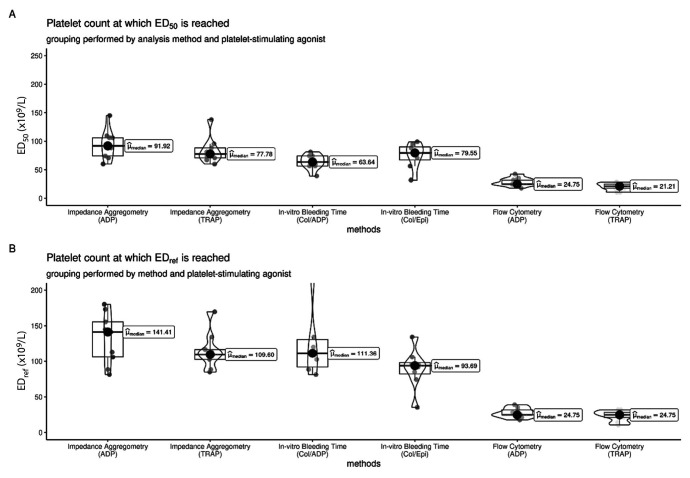
a) ED_50_ and b) ED_ref_ of IA, IVBT and FC (from left to right) calculated for each agonist separately. There was no significant difference between agonists used within any of the three methods, neither for ED_50_ nor ED_ref_. Both agonists used in FC had significantly lower ED_50_ and ED_ref_ values than any agonist used in IA and IVBT (P < 0.05). IA – impedance aggregometry. IVBT – *in-vitro* bleeding time. FC – flow cytometry. ED_50_ – median effective dose. ED_ref_ – the lower reference limit. ADP – adenosine diphosphate. TRAP – thrombin receptor- activating peptide. Col/ADP – collagen/ adenosine diphosphate. Col/Epi – collagen/ epinephrine.

Impedance aggregometry measurements showed significant impairment in platelet function with a platelet count below 100 x10^9^/L. Already with the first dilution step the median AUC fell by 17% for TRAP and 40% for ADP compared to the initial whole blood sample. In the lowest concentration group, results dropped to 3.4% (ADP and TRAP) of initial values (Figure 2a). The ED_50_ values for platelet stimulation with ADP and TRAP were 92 x10^9^/L and 78 x10^9^/L; the ED_ref_-platelet counts were 141 x10^9^/L, and 110 x10^9^/L, respectively (Figure 3). There was no significant difference between the stimulation with ADP or TRAP when comparing either ED_50_ or ED_ref_, although the stimulation with TRAP showed a (non-significant) tendency to require a lower platelet count until either threshold was reached.

#### In-vitro bleeding time

In samples with platelet counts below 100 x10^9^/L, an increase of CT by 32% for Col/Epi and 53% for Col/ADP could be shown compared to undiluted samples. In samples with platelet counts lower than 50 x10^9^/L, no occlusive clot was detected within 300s after Col/Epi stimulation, whereas with Col/ADP stimulation this was only reached at 30 x10^9^/L (Figure 2b). No significant difference in ED_50_ or ED_ref_ was determined between Col/ADP (64 x10^9^/L and 111 x10^9^/L, respectively) and Col/Epi (80 x10^9^/L and 94 x10^9^/L, respectively) (Figure 3).

### Flow cytometry

Flow cytometric assays showed consistent results even at low platelet counts. Samples with concentrations as low as 30-50 x10^9^/L still demonstrated 94% (ADP) and 96% (TRAP) of the initial whole blood P-selectin expression (Figure 2c). Stimulation with TRAP displayed a trend towards reaching the thresholds in both ED_50_ (21 x10^9^/L) and ED_ref_ (25 x10^9^/L) at lower platelet counts compared to stimulation with ADP (25 x10^9^/L each) but failed to reach statistical significance (Figure 3).

### Inter-assay comparison

**Figure 4:**
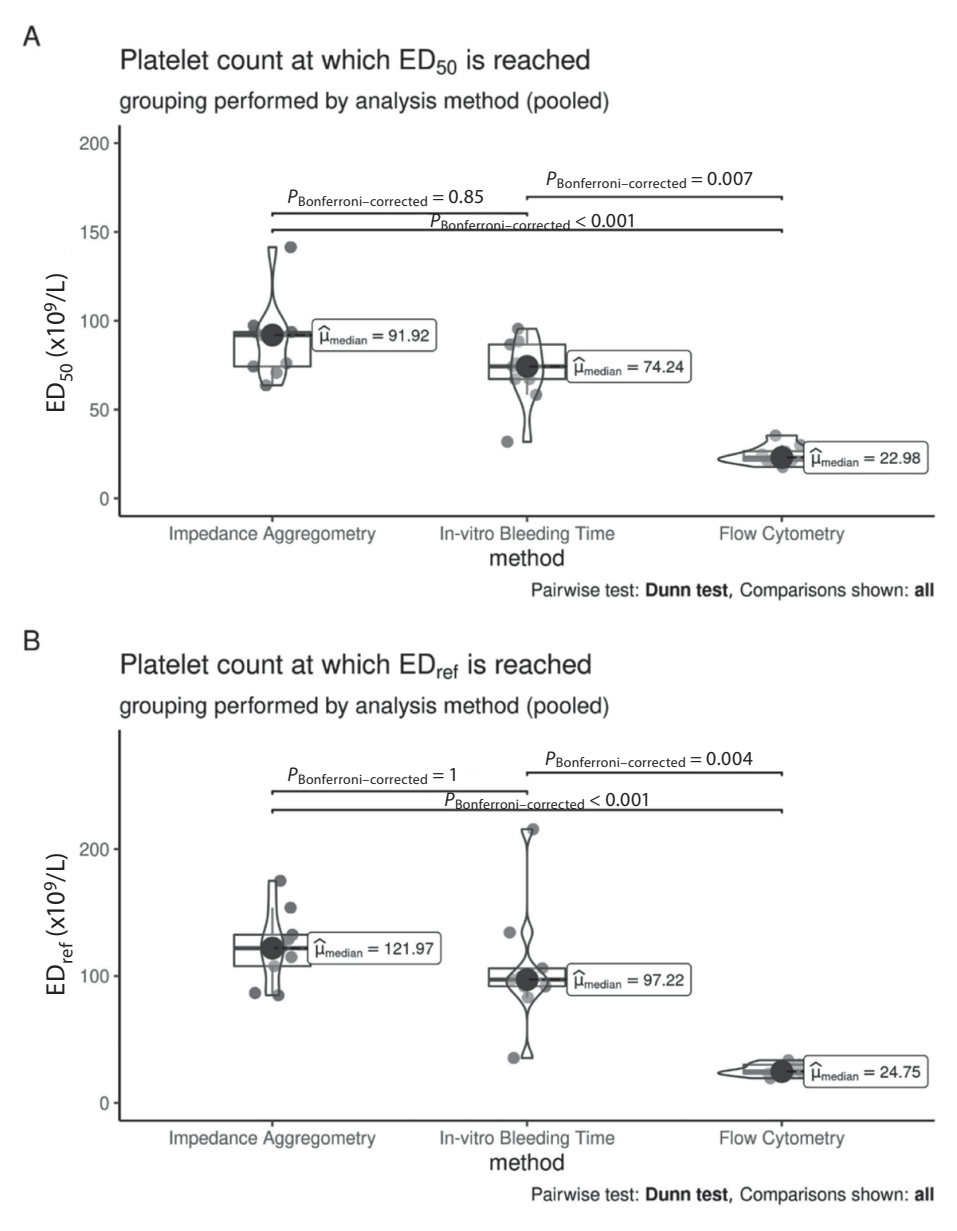
a) ED_50_ and b) ED_ref_ of IA, IVBT and FC (from left to right) calculated for each method after pooling data from both agonists respectively. IA – impedance aggregometry. IVBT – *in-vitro* bleeding time. FC – flow cytometry. ED_50_ – median effective dose. ED_ref_ – the lower reference limit.

When comparing the ED_50_ as well as the ED_ref_ of all three methods with each type of agonist, FC showed markedly lower limits compared to both IA and IVBT (adjusted P value < 0.05 each) (Figure 3). This also remained the case when pooling the different agonists and only comparing the methods (adjusted P values < 0.01 each) (Figure 4). Table 1 shows the numerical values of calculated ED_50_ and ED_ref_ from the separate and pooled analysis.

**Table 1:** Median values for median effective dose and reference range effective dose

	**Impedance aggregometry**		
	ADP	TRAP	ADP+TRAP pooled
Median ED_50_ (10^9^/L)	92	78	92
Median ED_ref_ (10^9^/L)	141	110	122
	***In-vitro* bleeding time**		
	Col/ADP	Col/Epi	Col/ADP + Col/Epi pooled
Median ED_50_ (10^9^/L)	64	80	74
Median ED_ref_ (10^9^/L)	111	94	97
	**Flow cytometry**		
	ADP	TRAP	ADP+TRAP pooled
Median ED_50_ (10^9^/L)	25	21	23
Median ED_ref_ (10^9^/L)	25	25	25
Values of median effective dose (ED_50_) and reference range effective dose (ED_ref_) shown for all three platelet function test methods (top to bottom), calculated for each agonist separately (left and middle column) as well as pooled analysis (right column).			

While IA displayed a trend toward higher platelet counts compared to IVBT, this effect did not prove to be statistically significant after correction for multiple testing, regardless if pooled or non-pooled data was used (Figures 3 and 4, respectively).

## Discussion

Impedance aggregometry and IVBT revealed incorrect tests results for platelet function when platelet count dropped below 100 x10^9^/L. We are the first to describe ED_50_ and ED_ref_ for effective inter-assay comparison of test results of two POC tests (IA, IVBT) and the gold standard (FC). When comparing the ED_50_ as well as the ED_ref_ of all three methods with different types of agonists, FC showed reliable results with considerably lower platelet counts, compared to both IA and IVBT. A further advantage of our study is the multi-step centrifugation technique we used to maintain physiological haematocrit value.

Boknäs and colleagues reconstituted blood samples of nine healthy volunteers to compare results of IA, light transmission aggregometry (LTA) and FC at four different platelet counts (200, 100, 50 and 10 x10^9^/L) (*11*). As in our study, TRAP and ADP were used as agonists for IA and results showed a similar decrease in measured platelet function with decreasing platelet count: this decrease was statistically significant starting from the first dilution level in both studies (100 x10^9^/L and < 100 x10^9^/L respectively). In contrast to Boknäs and colleagues, defined intervals in our study were narrower especially at thrombocytopenic levels between 50 and 100 x10^9^/L since we aimed to define lower limits of platelet count for use of POC tests in daily clinical practice. Even though in their study FC was the method, which was least affected by low platelet counts, the percentage of activated platelets decreased with very low platelet concentrations (<10 x10^9^/L) when using TRAP as an agonist. Stimulation with ADP was hardly affected by thrombocytopenia. In our study, P-selectin binding in FC was reduced in the lowest concentration group for both agonists (83% of initial whole blood values for ADP and 70% for TRAP). However, we cannot confirm the superiority of ADP over TRAP for low platelet counts in FC as our ED_50_- and ED_ref_ results suggest a slight trend towards the opposite. Although these differences were minor and not significant, they not only occurred in FC but in IA as well. Another study to investigate IA and FC at low platelet counts (median levels of 135, 107, 82 and 51 x10^9^/L) was published in 2016 by Tiedemann Skipper and colleagues (*10*). Their results demonstrated a significant, positive association of platelet counts (once below 200 x10^9^/L) and platelet aggregation for IA. In accordance with our findings, FC results also showed significant changes in the samples with the lowest platelet counts (median 51 x10^9^/L) but these remained only minor.

Stissing and colleagues described a similar relationship between IA results and decreasing platelet counts (200, 150, 100, 50 and 25 x10^9^/L) but did not account for concomitant dilution of haematocrit (*9*). In our study we carefully maintained a constant haematocrit throughout the dilution process. Failure to do so may constitute an important bias, since haematocrit is known to influence IA results (*20*, *21*).

Hanke and colleagues found a significant decrease of platelet function measured by IA for decreasing platelet counts (*3*). However, from the five created platelet concentrations only one group was below 100 x10^9^/L. This limits the relevance of the study, since thrombocytopenia above 100 x10^9^/L is rarely ever considered as clinically relevant.

The association of platelet counts and IVBT results was reported in 1996 by Kundu and colleagues, who reconstituted whole blood from six healthy volunteers with three different platelet counts (200, 100 and 50 x10^9^/L) and measured IVBT with the predecessor model PFA-100 (*12*). Unlike our study, assessment of platelet function was restricted to the Col/Epi test. They found a significant increase of CT between the highest and lowest platelet count (mean increase of 70% for samples with a platelet count of 50 x10^9^/L), which is similar to our results (82% increase between initial platelet count and 50–80 x10^9^/L). However, apart from including the Col/ADP test we examined the effect of thrombocytopenia more thoroughly by creating more and lower platelet counts. The following limitations have to be considered for our study: first, the sample size is limited which was due to the exploratory character of this study. The trend towards a better performance of TRAP in IA and FC when compared to ADP might have reached statistical significance with a bigger sample size. Second, results obtained in this *in-vitro* study might not reflect physiological conditions in-vivo in a one-to-one manner. Nevertheless, the modified technique we used to reconstitute thrombocytopenic blood samples allowed maintenance of haematocrit, which has previously been described to play an important role in platelet function testing. Third, platelets might have been activated during reconstitution of blood samples, especially the residual platelets in PPWB, which were subjected to the most intensive manipulation. Hence, we aimed to minimize preanalytical confounders by following standardized protocols in sample processing. Also, since we focused on intra-individual analyses, any remaining platelet pre-activation should not have affected the comparison between methods. Fourth, in order to complete tests within an acceptable time following venepuncture, the number of agonists was limited. This is why the conclusions drawn for IA and FC can only be applied to TRAP and ADP. Fifth, IVBT results are dependent on von Willebrand Factor levels and activity, which were not evaluated in the study population. Although this might have influenced IVBT test results the intra-individual analysis should have mitigated an eventual confounding effect. Finally, two volunteers with initial platelet counts in the thrombocytopenic range must be mentioned (131 x10^9^/L each). However, these values were still well within the limits of our undiluted samples (> 100 x10^9^/L) and initial platelet function was normal.

In summary, IA and IVBT spuriously measure reduced platelet function at platelet counts below 100 x10^9^/L. In FC, correct assessment of platelet function is warranted for samples with platelet counts > 30 x10^9^/L. In both FC and IA, ADP showed a tendency to require higher platelet count than TRAP for accurate results, although this trend did not reach statistical significance. The new model presented here for comparing different test methods by calculating their ED_50_ and ED_ref_ values proved to be robust and effective.
